# Positive feedback between ROS and *cis*-axis of PIASxα/p38α-SUMOylation/MK2 facilitates gastric cancer metastasis

**DOI:** 10.1038/s41419-021-04302-6

**Published:** 2021-10-22

**Authors:** Qian Wang, Ci Xu, Qiang Fan, Haihua Yuan, Xin Zhang, Biying Chen, Renjie Cai, Yanjie Zhang, Moubin Lin, Ming Xu

**Affiliations:** 1 Department of Oncology, Shanghai Ninth People’s Hospital, Shanghai Jiao Tong University School of Medicine, 280 Mohe Road, Shanghai, 201999 China; 2 Center for Clinical Research and Translational Medicine, Yangpu Hospital, Tongji University School of Medicine, 16 Boyang Road, Shanghai, 200090 China; 3 Shanghai Institute of Precision Medicine, Ninth People’s Hospital, Shanghai Jiao Tong University School of Medicine, 115 Jinzun Road, Shanghai, 200125 China

**Keywords:** Gastric cancer, Sumoylation

## Abstract

MAPK/p38 is an important mammalian signaling cascade that responds to a variety of intracellular or extracellular stimuli, such as reactive oxygen species (ROS), and participates in numerous physiological and pathological processes. However, the biological function of p38 in different tumors, and even at different stages of the same tumor, remains elusive. To further understand the regulatory mechanism of p38 and oxidative stress in the occurrence and development of gastric cancer, we report SUMOylation as a novel post-translational modification occurring on lysine 152 of MAPK14/p38α through immunoprecipitation and series of pull-down assays in vitro and in vivo. Importantly, we determine that p38α-SUMOylation functions as an authentic sensor and accelerator of reactive oxygen species generation via interaction with and activation of MK2 in the nucleus, and the ROS accumulation, in turn, promotes the SUMOylation of p38α by stabilizing the PIASxα protein. This precise regulatory mechanism is exploited by gastric cancer cells to create an internal environment for survival and, ultimately, metastasis. This study reveals novel insights into p38α-SUMOylation and its association with the intracellular oxidative stress response, which is closely related to the processes of gastric cancer. Furthermore, the PIASxα/p38α-SUMOylation/MK2 *cis*-axis may serve as a desirable therapeutic target in gastric cancer as targeting PIASxα, MK2, or a specific peptide region of p38α may reconcile the aberrant oxidative stress response in gastric cancer cells.

## Introduction

The cellular response to extracellular stimuli is, in part, dynamically mediated by a number of intracellular kinases and phosphatases [1, 2]. As members of discrete signaling cascades, mitogen-activated protein kinases (MAPKs) transduce signals from the cell membrane to the nucleus in response to diverse extracellular stimuli and regulate fundamental cellular processes [2], as well as pathological courses [3, 4]. The p38 mitogen-activated protein kinase (MAPK/p38) signaling pathway is involved in a variety of biological phenomena, especially oxidative stress (OS) and DNA damage, which are important causative factors of carcinogenesis [5–9].

Gastric cancer (GC) is the fifth most prevalent malignancy globally and is the third leading cause of cancer-related deaths [10]. *H. pylori* infection is the most common risk factor for GC, and stepwise progression of this cancer often leads to atrophic gastritis or intestinal metaplasia [10, 11]. Although chemotherapy can partially improve the survival and quality of life of patients, especially those with locally unresectable or metastatic GC, the median overall survival is still only around one year. This is because GC is frequently diagnosed at an advanced stage, wherein distant metastases may have occurred [10, 12]. Despite advances in the general understanding of the biology of GC, surgical or endoscopic resection remains the primary treatment strategy for GC [11].

The progression of GC is a complex process with multiple steps and stages, all of which involve DNA damage, excessive proliferation, apoptotic damage, and gene instability [10, 13]. Cellular signaling pathways altered in GC cells include the NF-κB, ERK/MAPK, ROS/ASK1/JNK, and MAPK/p38 pathways [14, 15]. Recent studies have shown that OS in cells is closely related to the occurrence and development of cancers [16]. Low concentrations of ROS in the tumor microenvironment can promote tumor glucose metabolism by inducing mitochondrial autophagy and activating signaling pathways to maintain the demand for high consumption of energy in tumors [16]. Persistently high ROS levels can cause oncogene activation, gene mutations, or chromosomal aberrations [17, 18]. Contrastingly, high ROS concentrations can also inhibit the growth and development of GC cells by inducing tumor cell apoptosis [19–21]. However, in-depth understanding of the effect of oxidative stress on the occurrence and development of gastric cancer is still in puzzle.

ROS are a class of oxidizing agents (including O_2_^-^, OH•, H_2_O_2_, NO, and ONOO^-^) that exert diverse effects on cellular components and play critical roles in maintaining tissue homeostasis [22–24]. Notably, high ROS levels can be reversed by corresponding cellular antioxidants (SOD, CAT, GSH-Px, SRX, TXN, vitamin C/E, and Zn^2+^) to prevent adverse consequences and to ensure an intracellular balance, which has a great impact on determining malignant progression of cancers [24–26]. In cancer cells, p38α is a key OS sensor in oncogenic transformation, and its activation can significantly inhibit HRAS^V12^-induced ROS accumulation by triggering apoptosis. Thus, p38α could function as a negative modulator of tumor initiation or malignant transformation under OS [9]. Interestingly, ROS was found to promote the proliferation of pancreatic ductal adenocarcinoma cells by inducing monocyte-to-myofibroblast trans-differentiation through the MAPK/p38 signaling pathway [27]. The activation of MAPK/p38 limits the lifespan of hematopoietic stem cells by increasing ROS levels [6]; however, this increase induced protective autophagy through regulation of autophagy-related genes in HeLa cells [28]. Therefore, the role of activation or inhibition of MAPK/p38 in promoting human cancer progression in the presence of OS largely depends on the tumor type or stage. However, there are still undiscovered regulatory mechanisms between OS and p38 that contribute to the occurrence and development of GC.

SUMOylation, one of the most common type of reversible posttranslational modifications (PTMs), is essential for the maintenance of genomic integrity, gene regulation, and intracellular signaling through dynamic regulation of protein structure, interaction, and spatial localization via attachment of small ubiquitin-like modifier (SUMO) to the lysine ε amino group or the SUMO interaction motif (SIM) [29, 30]. Additionally, SUMOylation levels on target proteins can be influenced by diverse intracellular or extracellular stimuli, such as growth factors [31, 32], inflammatory cytokines [33], kinase activities [34], hypoxia contexts [35], and ROS levels [34, 36, 37]. Thus, imbalance of the intracellular SUMO system or SUMOylation levels of functional substrates is closely related to disease development, including tumor initiation and cancer metastasis [30].

In this study, we reveal that p38α can be covalently SUMOylated which is associated with cellular OS. Therefore, understanding how OS responding and ROS production are precisely regulated during the development of GC and how p38α-SUMOylation is utilized to maintain the survival and metastasis of tumor cells under the ROS circumstance is of great clinical significance for the development of new strategies for the prevention and treatment of this cancer disease.

## Materials and methods

### SUMOylation assays

SUMOylation assay was performed according to the three different methods as described in previous reports [38–40]. Detailed procedures are available from supplemental materials.

### ROS detection

Intracellular ROS levels were measured with fluorescent probes 2ʹ,7ʹ-dichlorofluorescein diacetate (DCFH-DA) and CellROX Orange Reagent as per the manufacturer’s instructions. Detailed procedures are available from supplemental materials.

### Animal xenograft assay

After pre-selection (weight, size, or healthy state), four-week-old female nude mice (BALB/C) were divided into four groups randomly. The mice were bred in a specific pathogen-free condition in which the temperature was maintained at 22–25 °C and the humidity was set at 40%. Mice were injected with 1 × 10^6^ cells in 100 µL PBS via tail vein. A total of 5 mice in each group were monitored every three days and sacrificed at 45 days post injection. Lungs and livers of mice were collected and fixed with 4% paraformaldehyde for blind hematoxylin and eosin (H & E) and immunohistochemistry (IHC) staining analysis. The strong vimentin staining in IHC was considered the positive tumor loci. The experiment was approved by the Animal Ethics Committee of Shanghai Jiao Tong University School of Medicine.

### Statistical analysis

Each experiment was performed at least three times. The significance of the difference and variation between any two non-paired samples was calculated and tested with a *t*-test by using GraphPad Prism software (GraphPad Software, San Diego, CA, USA), in which *p* < 0.05(*) or <0.01(**) was considered statistically significant.

## Results

### SUMOylation occurs on p38α in the nucleus

**Fig. 1:**
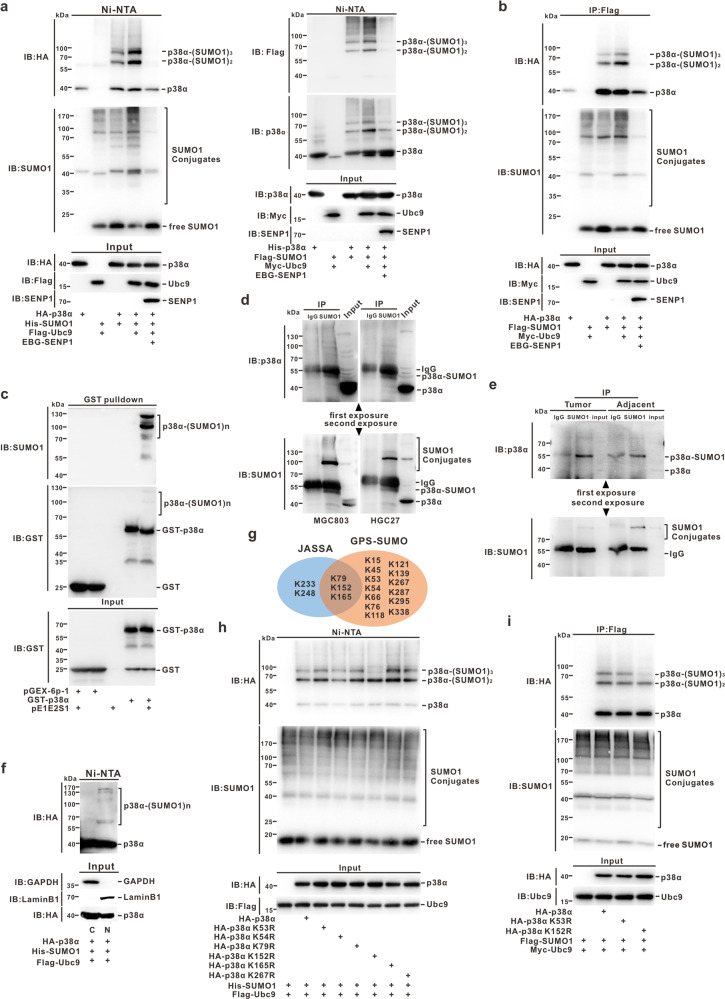
p38α can be SUMOylated at the major site K152 in the nucleus. **a**, **b** Lysis of HEK293T cells transfected with indicated plasmids was subjected to a subsequent Ni^2+^-NTA resin pull-down (**a**) or immunoprecipitated with mouse antiFLAG antibody (**b**), and exogenous SUMO1 modification of p38α was immunoblotted with the indicated antibodies. **c** Plasmid pGEX-6p-1/p38α was co-transfected with or without pE1/E2/SUMO1 plasmid into *E. coli* BL21. Immunoblotting was performed with anti-SUMO1 antibody after GST pull-down assay; the same membrane was stripped and blotted with anti-GST antibody. **d**, **e** Endogenous SUMOylation on p38α in GC cell lines (MGC803 and HGC27) (**d**), or in GC patient specimens (tumor and adjacent tissues) was analyzed with immunoprecipitation assay and detected with indicated antibodies. **f** After transfection with the indicated plasmids, HEK293T cells were extracted by the nuclear/cytosol fractionation kit, following which the lysates were pulled down by using the Ni^2+^-NTA resin and analyzed by immunoblotting. **g** The potential SUMOylation sites on p38α predicted by JASSA and GPS-SUMO software are shown in the pie chart. **h** The construct of pEF-5HA/p38α^WT^ or p38α mutants was respectively co-transfected with His-SUMO1 into HEK293T cells. Cells were harvested 48 h after transfection and the lysates were analyzed by immunoblotting after pulling down with the Ni^2+^-NTA resin. **i** The construct, pEF-5HA/p38α^WT^ or p38α-K152R, was co-transfected with FLAG-SUMO1 into HEK293T cells. Cells were harvested at 48 h after transfection and lysates were analyzed by immunoblotting after immunoprecipitation with anti-FLAG antibody.

To determine whether p38α was SUMOylated, the Ni^2+^-NTA pull-down assay was conducted. The results showed that His-SUMO1 conjugated with HA-p38α (Fig. 1a, left panel), or Flag-SUMO1 conjugated with His-p38 (Fig. 1a, right panel), and the reaction could be enhanced by SUMOylation E2 enzyme Ubc9 or attenuated by de-SUMOylation enzyme SENP1 in HEK293T cells, respectively (Fig. 1a and Fig. S1a). A similar result was observed by an immunoprecipitation assay performed according to a previous report [40] (Fig. 1b and Fig. S1b). Furthermore, a GST pulldown assay using an *E. coli* prokaryotic expression system confirmed that GST-p38α was strongly SUMOylated upon co-transformation with pE1/E2/SUMO1 (Fig. 1c and Fig. S1c). To identify endogenous SUMOylation on p38α, lysates of MGC803 and HGC27 cell lines were analyzed by an immunoprecipitation assay. Endogenous SUMO1modified p38α was only successfully detected by p38α antibody in the complexes immunoprecipitated with the SUMO1 antibody but not in those immunoprecipitated with the normal IgG (Fig. 1d). A similar phenomenon was also observed by analyzing the lysates of tumor and adjacent surgical tissues from GC patients (Fig. 1e). Moreover, the SUMOylation on p38α occurred only in the nucleus according to Ni^2+^-NTA pull-down assay by using the extracted lysates of nuclear/cytosol fractionation (Fig. 1f). Collectively, these results demonstrated that p38α can be SUMOylated in vitro and in vivo, and this modification takes place only in the nucleus.

### SUMO1 is mainly attached to K152 in p38α

**Fig. 2:**
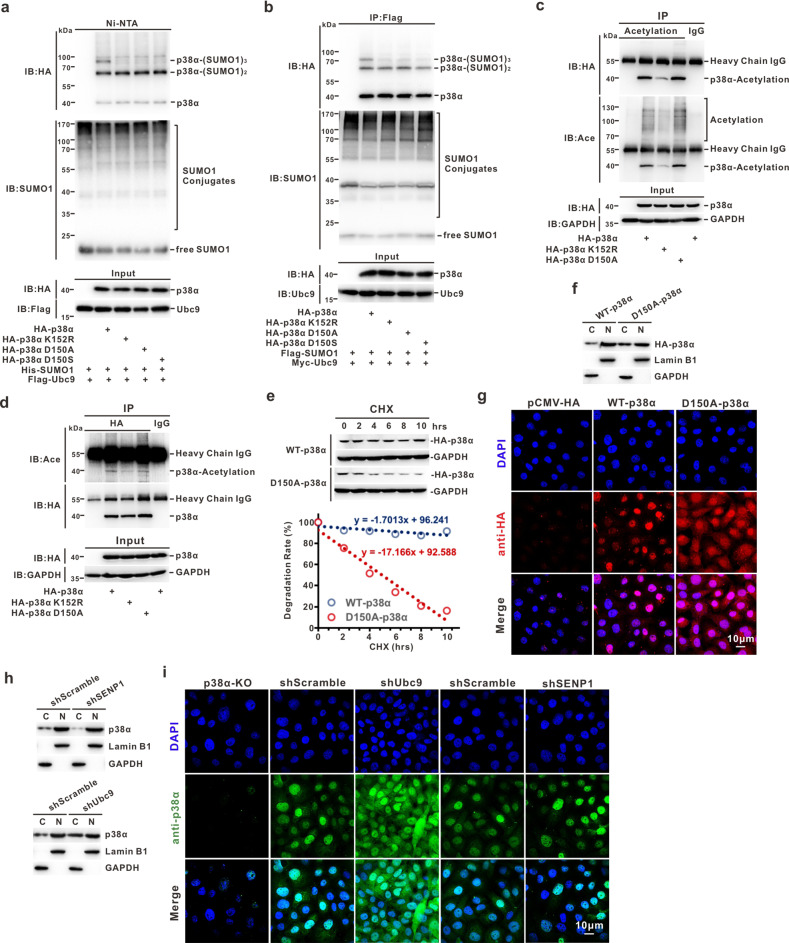
SUMOylation ensures protein stability and nuclear translocation of p38α. **a**, **b** The constructs containing pEF-5HA/p38α-WT, -K152R, -D150A, or -D150S were co-transfected with indicated plasmids into HEK293T cells. Cells were harvested at 48 h after transfection and the lysates were analyzed by immunoblotting after a Ni^2+^NTA resin pull-down (**a**) or immunoprecipitated with mouse anti-FLAG antibody (**b**). **c**, **d** Exogenous HA-p38α-WT, -K152R, or -D150A were transfected in HEK293T cells, and cell lysates were subjected to immunoprecipitation with antibody against ACE-lysine (**c**) or HA (**d**), immunoblotted with anti-HA or anti-ACE-lysine antibody, respectively. **e** Cells stably expressing HA-tagged p38α-WT or p38α-D150A were treated with 100 μM CHX for time periods, and then cell lysates were subjected to immunoblotting analysis with the HA or GAPDH antibody (upper panel). The degradation rate was quantified as a line diagram (lower panel). **f**, **g** HeLa cells transfected with HA-tagged p38α-WT or p38α-D150A were extracted by the nuclear/cytosol fractionation kit, and the lysates (total 50 µg/sample) were subjected to immunoblotting with indicated antibodies (**f**) and immunofluorescence staining with primary mouse anti-HA antibody (1:200) and Alexa Fluor 568 labeled secondary antibody (1:200). The nuclei were counterstained with DAPI (**g**). **h**–**i** The stable HeLa cell lines with shRNAscramble, -SENP1 or -Ubc9 were extracted by the nuclear/cytosol fractionation kit, and lysates (total 50 µg/sample) were subjected to immunoblotting with indicated antibodies (**h**) and immunofluorescence staining using the primary rabbit anti-p38α antibody (1:200) and Alexa Fluor 488 labeled secondary antibody (1:200). Cell nuclei was counterstained with DAPI (**i**). The images were captured by Leica confocal microscopy, scale bar: 30 µm (**g**, **i**).

According to the prediction by JASSA (http://www.jassa.fr) and GPS-SUMO 2.0 Online Service (http://sumosp.biocuckoo.org/) (Fig. 1g and Fig. S1d, e), a total of six potential SUMOylation sites were selected within p38α and mutated by replacing R (arginine) with K (lysine). Wild-type (WT) or mutant HA-p38α (K53R, K54R, K79R, K165R, K152R, K267R) constructs were co-transfected with His-SUMO1 into HEK293T cells for the Ni^2+^-NTA pull-down assay. We found that the K152R mutation reduced the SUMOylation level of p38α, but the other mutants exhibited no or weak changes compared with those of p38α^WT^ (Fig. 1h). Immunoprecipitation assay also confirmed that K152 was the major SUMO1 modification site in p38α (Fig. 1i). However, K152 has already been reported as the acetylation site in p38α [41]. To exclude the impact of acetylation on p38α in follow-up experiments, we tried to disrupt the canonical consensus motif of ψ-K-x-D/E (directionless; ψ, a hydrophobic amino acid; x, any amino acid residue; D_150_LK_152_P in p38α) with D150A or D150S for exclusively affecting SUMOylation, without potentially impairing acetylation of p38α. Ni^2+^-NTA pull-down and immunoprecipitation assays showed that D150A or D150S, both similar to K152R, could reduce p38α-SUMOylation level (Fig. 2a, b), but barely affected its acetylation level (Fig. 2c, d). Together, these results suggested that K152 is a major SUMO1 modification site in p38α, and p38α variants harboring a mutation of D150A or D150S can be further used for analyzing the exact function of SUMOylation in p38α, without interference or ‘noise’ derived from acetylation.

### SUMOylation ensures protein stability and nuclear translocation of p38α

SUMOylation and ubiquitylation are largely distinct and independent, but there is substantial evidence of cross-talk between them. SUMOylation can affect protein stability as well as substrate translocation [42]. Keeping this in mind, HA-p38α^WT^ and HA-p38α^D150A^ were introduced into HeLa cells, respectively, and a pulse-chase assay showed that the protein half-life of HA-p38α^D150A^ was significantly decreased as compared to that of HA-p38α^WT^ after treatment with cycloheximide (CHX) (Fig. 2e). Strikingly, analysis of the nuclear-to-cytosolic ratio revealed that a fraction of HA-p38α^WT^ translocated to the nucleus, but HA-p38α^D150A^ tended to localize in the cytoplasm (Fig. 2f). This result was corroborated by that of the immunofluorescence assay (Fig. 2g). To confirm that SUMOylation could affect the subcellular localization of p38α, HeLa cell lines with lower or higher SUMOylation conditions were constructed by depletion of Ubc9 or SENP1, respectively (Fig. S2a). Similar to the above results, the nuclearto-cytosolic ratio of endogenous p38α was decreased in HeLa-shUbc9 cells and increased in HeLa-shSENP1 compared with that in control cells (Fig. 2h). Consistently, the immunofluorescence assay also showed a similar phenomenon (Fig. 2i). Taken together, these data demonstrated that SUMOylation of p38α not only maintains protein stability but also ensures its nuclear translocation.

### p38α-SUMOylation facilitates GC metastasis in vitro and in vivo

**Fig. 3:**
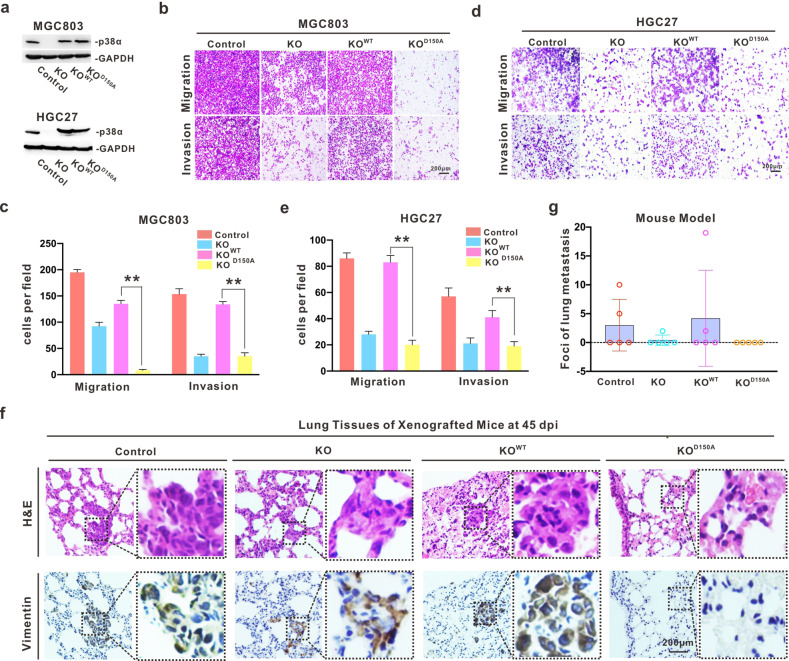
p38α-SUMOylation facilitates GC metastasis in vitro and in vivo. **a** Endogenous MAPK14 mRNA was disrupted for inducing p38α-translationdeficiency (KO) in both MGC803 and HGC27 cell lines using the CRISPR-Cas9 system, and exogenous HA-p38α^WT^ or HA-p38α^D150A^ were re-introduced into MGC803/MAPK14^KO^ and HGC27/MAPK14^KO^ cells by lentiviral infection. The expression of endogenous and exogenous p38α proteins in those cell lines was analyzed by immunoblotting. **b**–**e** Migrated and invaded cells was stained with crystal violet and captured for this presentation (**b**, **d**). The number of positive cells that went through the out-bottom of inner chambers was statistically presented as the mean ± S.D. **p < 0.01 (**c**, **e**). **f**–**g** The lungs of xenografted mice were collected for H & E staining and immunoblotting with anti-vimentin antibody at 45 days after tail intravenous injection with the indicated HGC27 stable cell lines. The representative images are presented, scale bar: 200 µm (**f**). The number of lung metastatic loci in each nude mouse is shown in the dot plot (**g**).

To investigate the cellular function performed by p38α-SUMOylation in GC, we established p38α, but not p38β, knock-out cell lines MGC803/p38α^KO^ and HGC27/p38α^KO^ with the CRISPR-Cas9 system, and the resultant colonies were validated by Western blotting and genotyping (Fig. S2b–d). Next, we re-introduced p38α^WT^ or p38α^D150A^ into MGC803/p38α^KO^ and HGC27/p38α^KO^ cell lines by lentiviral infection (Fig. 3a). A series of phenotypic analyses revealed that p38α^D150A^, similar to p38α knockout, significantly inhibited cell migration and invasion compared to p38α^WT^ in both MGC803 and HGC27 cells (Fig. 3b–e). However, there was no distinguished difference in cell proliferation between these cells (Fig. S2e, f). In the xenograft model, the specified cell lines were intravenously injected into nude mice, and the lungs of necropsied mice were collected for H&E staining and IHC detection with human vimentin antibody at 45 days post injection. p38α^KO^ significantly decreased the tendency for pulmonary metastasis in GC cells, which was restored by re-introducing p38α^WT^, but not p38α^D150A^ (Fig. 3f, g). Taken together, p38α-SUMOylation can facilitate the metastasis processes of GC in vitro and in vivo, at least pulmonary metastasis.

### Positive feedback between p38α-SUMOylation and ROS facilitates metastasis of GC cells

**Fig. 4:**
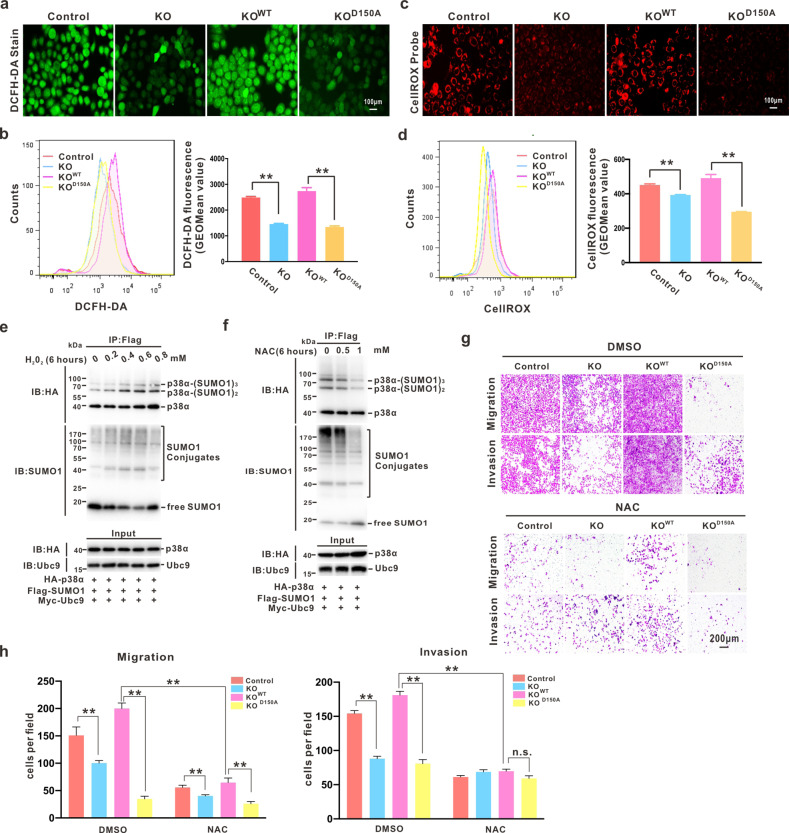
Positive feedback between p38α-SUMOylation and ROS facilitates the metastatic property of GC cells. **a**–**d** ROS levels in HGC27/MAPK14^KO^ cells with re-supplied p38α^WT^ and p38α^D150A^ were determined by DCFH-DA probe and CellRox Orange for fluorescence microscopic observation (**a**, **c**) and flow cytometry analysis (**b**, **d**, left panels). The statistical data are presented as mean ± S.D., **p < 0.01 (**b**, **d**, right panels). **e**–**f** HEK293T cells co-transfected with FLAG-SUMO1, Myc-Ubc9, and HA-p38α-WT were treated with gradient concentrations of H_2_O_2_ or NAC for 6 h, respectively, before harvesting. The cell lysates were immunoprecipitated with anti-FLAG antibody for SUMOylation assay and Western blotting. **g**–**h** The indicated HGC27 stable cell lines were treated with DMSO or NAC (1 mM), respectively, for both migration and invasion assays (**g**). The data are presented as the mean ± S.D. **p < 0.01 (**h**).

Since p38α is a critical OS sensor, we wondered whether p38α SUMOylation could affect ROS generation in GC. Staining with either the DCFH-DA fluorescent probe or CellROX Orange reagent was performed in HGC27 cell lines. Both fluorescence microscopic observations and flow cytometry analysis revealed that deSUMOylated p38α (p38α^D150A^) could not efficiently induce ROS in GC cells like p38α^WT^ (Fig. 4a–d). The primary ROS, H_2_O_2,_ was also able to significantly promote the SUMOylation level of p38α (Fig. 4e, lanes 1–4). However, excess levels of H_2_O_2_ did not further enhance the SUMOylation process (Fig. 4e, lane 5), which could be associated with an unknown self-protective mechanism in tumor cells. In contrast, an antioxidant N-acetyl cysteine (NAC) was employed to confirm that ROS could positively induce SUMOylation event on p38α (Fig. 4f). According to the migration and invasion assay in HGC27 stable cell lines, NAC significantly suppressed the p38α-SUMOylation-mediated metastatic properties of GC cells (Fig. 4g, h). Therefore, these data suggested that SUMOylation is critical for p38α to function as a sensor to generate ROS, and positive feedback between p38α-SUMOylation and ROS may contribute to the GC metastatic processes.

### MK2 activation is essential for p38α-SUMOylation to mediate OS response in GC cells

**Fig. 5:**
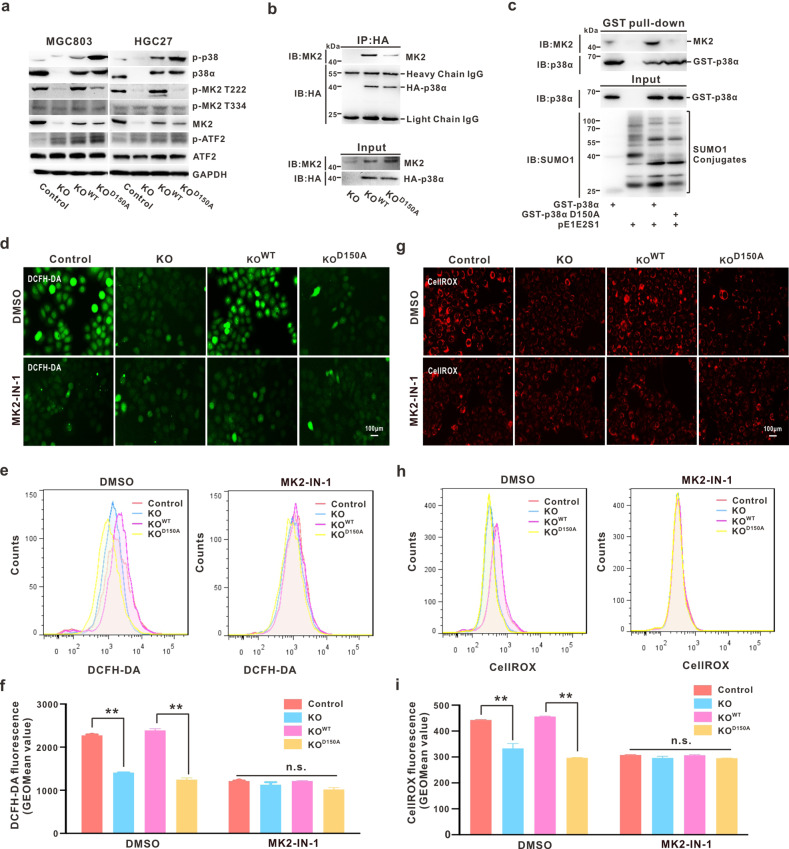
MK2 activation is essential for p38α-SUMOylation to mediate OS response in GC cells. **a** p38-associated proteins were detected by Western blotting in indicated cell lines generated from both MGC803 and HGC27. **b** The interaction between HA-p38α^WT or D150A^ and MK2 was detected by co-immunoprecipitation analysis in MGC803/MAPK14^KO^ cells. **c** MGC803 cell lysates were incubated with purified GST-p38α^WT^ or p38α protein overnight at 4 °C and subjected to immunoblotting analysis. **d**–**i** ROS levels in indicated HGC27 stable cell lines after treatment with MK2-IN-1 hydrochloride (10 mM) for 24 h were determined by DCFH-DA probe (**d**–**f**) and CellRox Orange (**g**–**i**) for fluorescence microscopy (**d**, **g**) and flow cytometry analysis (**e**, **h**). The data are presented as mean ± S.D. **p < 0.01 (**f**, **i**).

To explore the mechanism through which p38α-SUMOylation mediates the OS response, which subsequently results in GC metastasis, we first focused on the closely related substrates of p38α: ATF2 and MK2. Western blotting demonstrated that the level of phosphorylated MK2 (pMK2, mainly at Thr222) in both MGC803 and HGC27 cells was significantly and completely recovered by re-introducing p38α^WT^ into p38α^KO^ cells, but not by introducing p38α^D150A^ (Fig. 5a). The levels of pATF2, as well as pp65, pSTAT1, and pSTAT3 (the other substrates of p38α), showed no significant difference between the p38α^D150A^ and p38α^WT^ groups (Fig. 5a and Fig. S3a). Of note, the MK2 protein level was significantly decreased upon endogenous p38α knockout (Fig. 5a), which is probably due to the fact that p38α affects the transcription or protein stability of MK2 (not detected). Moreover, p38α was hyperactivated when p38α^D150A^ was re-introduced compared to p38α^WT^, but still could not efficiently activate MK2 (Fig. 5a) in both MGC803 and HGC27 cell lines. To determine the basis for altered pMK2 activation, we conducted a co-immunoprecipitation assay and found that the interaction between p38α^D150A^ and MK2 was significantly weakened (Fig. 5b). As mentioned above, the SUMOylation of p38α occurred in the nucleus (Fig. 1f), and de-SUMOylation would affect the translocation of p38α (Fig. 2f–i). To eliminate the possibility of translocation causing an alteration in interactions, the GST pull-down system in *E. coli* was employed. Results showed that the binding action between p38α and MK2 was largely dependent on the SUMOylation status of p38α (Fig. 5c).

As MK2 has been reported to play an important role in mediating ROS generation in neutrophils [43], we analyzed the role of MK2 in p38α-SUMOylation and OS response in GC. By blocking MK2 activity by MK2-IN-1 hydrochloride (MK2 specific inhibitor) (Fig. S3b), the extreme intracellular ROS generated in p38α^WT^ cells was reduced to almost the same level as that in p38α^D150A^ or p38α^KO^ cells, as indicated by DCFH-DA probe staining (Fig. 5d) and flow cytometry analysis (Fig. 5e, f). In agreement with this result, CellROX staining and analysis also presented a similar outcome (Fig. 5g–i). Taken together, these results elucidated that MK2 activation is essential for p38α-SUMOylation, which further mediates the OS response in GC cells.

### ROS can stabilize PIASxα to promote SUMOylation on p38α

**Fig. 6:**
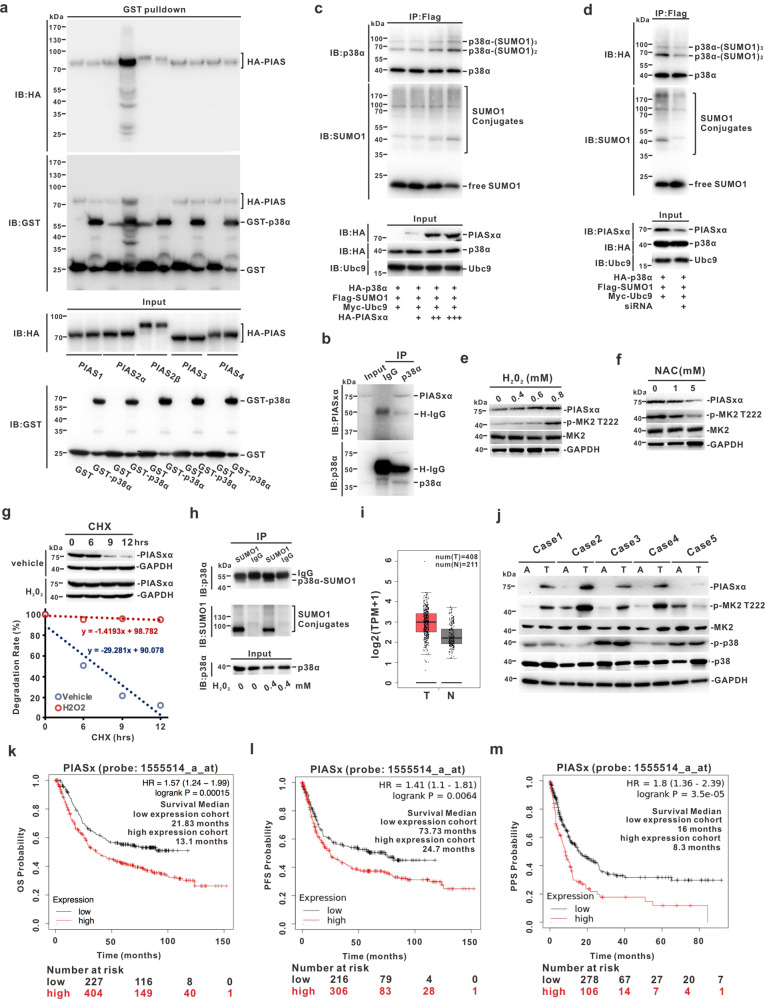
ROS can stabilize PIASxα to promote p38α-SUMOylation. **a** HEK293T cells were transfected with HA-tagged PIASs for 48 h, and cell lysates were incubated with purified GST-p38α protein overnight at 4° C and subjected to immunoblotting analysis. **b** Immunoprecipitation was conducted to detect the endogenous interaction between PIASxα and p38α in HGC27 cells. **c** HEK293T cells were co-transfected with FLAG-SUMO1, Myc-Ubc9, HA-p38α-WT, and gradient HAPIASxα for 48 h; cell lysates were immunoprecipitated with anti-FLAG antibody for the SUMOylation assay. **d** HEK293T cells were transfected with FLAG-SUMO1, Myc-Ubc9, HA-p38α-WT with or without PIASxα-siRNA, and cell lysates were immunoprecipitated for the SUMOylation assay. **e**, **f** HGC27 cells were treated with a gradient concentration of H_2_O_2_ (**e**) or NAC (**f**) for 6 h before harvesting, and the cells were lysed for Western blotting. **g** HGC27 cells stimulated by H_2_O_2_ or vehicle were treated with 50 μM CHX for indicated times and cell lysates were subjected to immunoblotting (upper panel). The degradation rate is represented as a line diagram (lower panel). **h** HGC27 cells were treated with or without H_2_O_2_ (0.8 mM) for 6 h and were harvested for detecting the SUMOylation level of endogenous p38α by immunoprecipitation. **i** GEPIA online database was used to show the mRNA level of PIASx in GC tissue and normal gastric tissues (T: tumor; N: normal). **j** Immunoblotting for determination of PIASxα, pp38, and pMK2 levels in 5 pairs of GC tissues and adjacent normal tissues (A: adjacent normal gastric tissues; T: GC tissues). **k**–**m** The survival probabilities were predicted by the online database. OS: overall survival (**k**), PFS: progression-free survival (**l**), or PPS: post-progression survival (**m**). Patients with high or low PIASx (PIASxα and PIASxβ) expression are indicated in red or black line, respectively. HR hazard ratio. The *p*-value was calculated by a log-rank test.

SUMOylation, similar to ubiquitylation, is a reversible modification mediated by E3 SUMO-protein transferases (PIASs) and de-SUMOylation enzymes (SENPs) [42]. To determine how ROS affects p38α-SUMOylation, a total of five PIASs (PIAS1, PIASxα, PIASxβ, PIAS3, PIAS4) were used for the GST pull-down assay and their ability to bind to p38α was analyzed. Results showed that PIASxα was more likely to interact with p38α (Fig. 6a). The endogenous interaction between PIASxα and p38α in HGC27 cells was also confirmed by the immunoprecipitation assay (Fig. 6b). To identify whether PIASxα could promote SUMOylation on p38α, the immunoprecipitation assay was performed. Results showed that p38α-SUMOylation was gradient enhanced following PIASxα transfection (Fig. 6c). In contrast, ablation of PIASxα using siRNA reduced the SUMOylation level of p38α (Fig. 6d). All these indicated that PIASxα is an authentic and direct regulator of p38α.

**Fig. 7:**
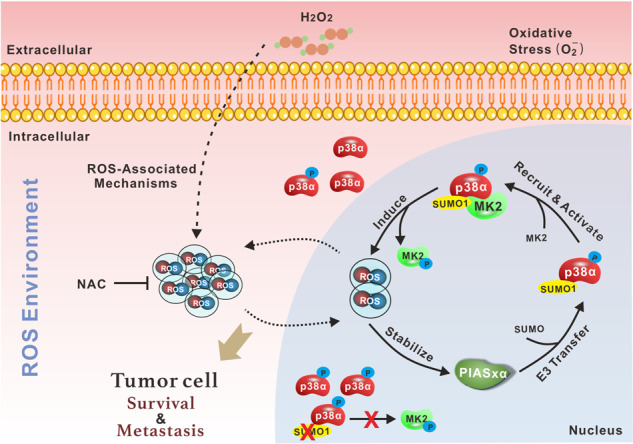
Graphical abstract. ROS, as a double-edged sword, possess both pro- and anti-tumorigenesis properties, which depends primarily on intracellular ROS level and tumor type. In gastric cancer, the oxidative stress sensor p38α can undergo SUMO1 modification to facilitate its nuclear translocation, and p38α-SUMOylation is essential for the activation of MK2 in the nucleus, thereby mediating ROS generation in cells. Meanwhile, an appropriate amount of ROS can stabilize SUMO E3 ligase PIASxα and further promote the SUMOylation of p38α, thus forming a positive feedback between ROS and *cis*-axis PIASxα/p38α-SUMOylation/MK2. This specific regulatory mechanism is exploited by gastric cancer cells to maintain initial tumor cell survival and further metastasis.

Moreover, H_2_O_2_ or NAC enhanced the SUMOylation process on p38α (Fig. 4e, f). We next measured the protein level of PIASxα. Surprisingly, ROS could positively regulate PIASxα only at the protein level (Fig. 6e, f), but not at the mRNA level (Fig. S3c). Pulse-chase assay revealed that H_2_O_2_ could significantly stabilize the PIASxα protein in GC cells (Fig. 6g and Fig. S3d). Besides, MK2 activity was *cis*-regulated following H_2_O_2_ stimuli or NAC treatment in GC cells (Fig. 6e, f, Fig. S3e, f), which was consistent with that of HEK293T cells (Fig. S3g, h). Similarly, the endogenous p38α-SUMOylation level was significantly enhanced by H_2_O_2_ (Fig. 6h). Clinically, the GEPIA database (http://gepia.cancer-pku.cn) showed that the expression of PIASx (including PIASxα and PIASxβ) was significantly higher in gastric tumor samples than in normal tissues (Fig. 6i), and this finding was confirmed at the protein level, together with the activities of p38α and MK2, using fresh surgical specimens from patients (Fig. 6j). Moreover, the survival curve revealed that higher PIASx expression predicted poor survival probability, among overall, progression-free or post-progression (Fig. 6k–m). In conclusion, these data demonstrated that the oncogene PIASxα is stabilized by ROS and it promotes the SUMOylation process of p38α, which creates a form of positive feedback between ROS and the *cis*-axis PIASxα/p38α-SUMOylation/MK2 to facilitate the metastasis of GC (Fig. 7).

## Discussion

MAPK/p38 is an essential component of the MAPK signaling pathway and plays a critical role in the signaling cascades triggered by extra- or intra-cellular stimuli such as inflammatory cytokines or physical stress resulting in direct activation of transcription factors [2]. Prior to the present study, only three PTMs (i.e., phosphorylation, acetylation, and ubiquitination) of p38α have been reported, and each modification has a significant impact on the activity and function of p38α in cells (Fig. S4) [44]. Moreover, targeting MAPK/p38 has shown promising therapeutic potential in multiple cancers [44–46]. The poly-SUMO2 chain can noncovalently interact with p38 to enhance the nuclear transfer of pp38 upon *H. pylori* infection, but the function of SUMO-modified p38 is remains unclear [47]. Herein, we identified that SUMO1 modification mainly occurs at the K152 position of p38α. To exclude the impact of K152-acetylation in p38α, the alternative variant p38α^D150A^, which reduced SUMOylation without affecting the acetylation of p38α, was used for the functional illustration of p38α-SUMOylation (Fig. 2a–d). According to our results, p38α-SUMOylation can promote the metastasis of GC through activation of MK2, which induced ROS generation in vitro and in vivo (Figs. 3 and 5). Compared with p38α^KO^ cells, cells harboring SUMOylation-deficient p38α presented metastasis suppression phenotypes (Figs. 3b–g and 4g, h), as well as a reduced capability for producing ROS (Figs. 4a–d and 5d–i). We also observed that ROS can promote the SUMOylation of p38α (Fig. 4e, f), creating a positive feedback loop.

Several studies have shown that the p38/MK2 pathway is closely related to the OS response [43, 48, 49]; however, we revealed that SUMOylation is required by p38α to activate MK2 in the nucleus (Fig. 5a–c) and to generate ROS in GC cells (Fig. 5d–i), and this requirement appears to be independent of p38 phosphorylation levels. SUMOylation-deficient p38α was unable to enter into or remain in the nucleus for MK2 activation, despite p38α hyperactivation (Fig. 5a, lane 4 in each panel). By contrast, the difference in ROS levels under p38α-SUMOylation and de-SUMOylation could be abolished by an MK2 inhibitor (Fig. 5d–i). Hence, p38α-SUMOylation-mediated ROS generation is largely dependent on MK2 activity.

This study also explored how ROS affects the SUMOylation of p38α. ROS could significantly stabilize PIASxα (Fig. 6e–g) and specifically interact with p38α in vivo and in vitro to promote its SUMOylation (Fig. 6a–d). PIASxα and PIASxβ share the same promoter and are closely associated with the MAPK/p38 signaling pathway [50, 51]. MAPK/p38 activation can stabilize PIASxβ and promote its transcription [50]. The phosphorylation of PIASxα mediated by p38β2 is deemed to dampen the activation of Elk1 by maintaining Elk1 SUMOylation and HDAC2 recruitment under anisomycin stimulation [50]. However, we observed that the stability of PIASxα is also related to the ROS level and positively correlates with the status of MK2 phosphorylation or p38α-SUMOylation level (Fig. 6e, f, h).

To be mentioned, there still are some shortcomings in this work, such as that the un-SUMOylated HA-p38α in control samples was prone to attach to the agarose or Ni-NTA resin non-specifically according to the Western blot analysis even though we have tried our best to solve this issue through optimizing the lysis and wash buffer (Figs. 1a, b and 2a, b). More importantly, the residual deSUMOylation enzymes, especially in the non-denatured RIPA buffer for immunoprecipitation assay, could inevitably remove the SUMO1 conjugates from the SUMOylated-p38α and lead to the detectable events of un-modified species during the experimental operation (Figs. 1a, b, i, 2a, b, 4e, f, and 6c, d). In addition, we have not succeeded in the reciprocal immunoprecipitation with HA antibody for p38α-SUMOylation assay, which could be affected by the poor immunoprecipitation efficiency of antibody for the HA-p38α-SUMOylation species (Fig. 1b).

In summary, we revealed that p38α can be utilized by GC cells for PIASxα-mediated SUMOylation in the nucleus to enhance MK2 activity and generate moderate levels of ROS for survival and to facilitate metastasis. Additionally, ROS can stabilize PIASxα to create a positive feedback loop between ROS and the *cis*-axis of PIASxα/p38α-SUMOylation/MK2. However, how ROS regulates the stability of PIASxα still need to be further researched, in which the specific ubiquitin E3 ligase or deubiquitinase could be the key regulators of PIASxα upon OS in GC. Beyond that, there must be certain unknown negative feedback mechanism to restrain PIASxα/p38α-SUMOylation/MK2 signal under the high ROS environment to resist the damage of high ROS on tumor cells. Here, our findings provide novel insights into the role of p38α in GC and provide potential therapeutic strategies for GC treatment, as targeting PIASxα, MK2, or a specific peptide region of p38α may resolve aberrant intracellular OS in GC cells and impede metastasis. Importantly, more attention should be paid to investigate the precise regulation mechanism of oxidative stress in various tumor diseases for therapeutic strategy making.

## Supplementary information

Supplemental Materials and Methods

Figure S1

Figure S2

Figure S3

Figure S4

Supplementary Figure Legends

## Data Availability

All the datasets generated and/or analyzed during this present study are available from the corresponding author on reasonable request.
