# Supplementary material for: Positive feedback between ROS and cis-axis of PIASxα/p38α-SUMOylation/MK2 facilitates gastric cancer metastasis
Source: Cell Death Dis. 2021 Oct 22;12(11):986. doi: 10.1038/s41419-021-04302-6 (PMC8536665; doi:10.1038/s41419-021-04302-6)
Supplement: Supplementary file 1 — Supplemental Materials and Methods [file 41419_2021_4302_MOESM1_ESM.docx]

# **Supplementary Materials and Methods**

## Cell culture and cell lines

Human gastric cancer (MGC803, HGC27), human cervical carcinoma (HeLa), and human embryonic kidney 293T (HEK293T) cell lines were purchased from Cell Bank of Type Culture Collection of Chinese Academy of Sciences (Shanghai, China). Both MGC803 and HeLa cell lines were cultured in Roswell Park Memorial Institute (RPMI) 1640 medium (Hyclone, UT, USA) with 10% fetal bovine serum (BioSun, Shanghai, China), 100 mg/mL streptomycin, 100 U/mL penicillin (Hyclone, UT, USA), and 1% GlutaMAX (Gibco, GI, USA). HGC27 and HEK293T cell lines were cultured in high-glucose Dulbecco’s Modified Eagle Medium (DMEM) (Hyclone, UT, USA) with the same supplements mentioned above. All cell lines used in this project were authenticated by STR identification and tested for mycoplasma contamination.

## Antibodies and reagents

Antibodies against rabbit HA-tag (C29F4, #3724), pp38 (Thr180/Tyr182) (D3F9, #4511), MK2 (D1E11, #12155), pMK2 (Thr334) (27B7, #3007), pMK2 (Thr222) (9A7, #3316), STAT1 (#9172), pSTAT1 (D3B7, #8826), STAT3 (124H6, #9139), pSTAT3 (Tyr705) (D3A7, #9145) p65 (D14E12, #8242), and pp65 (Ser536) (93H1, #3033) were all purchased from Cell Signaling Technology. SUMO1 antibody (PA5-81057) was obtained from Invitrogen (Carlsbad, CA, USA). IgG fraction monoclonal mouse antirabbit IgG (light chain-specific) was purchased from Jackson ImmunoResearch (West Grove, PA, USA). p38 (ab170099), goat antirabbit IgG H & L Alexa Fluor 568 (ab175471), goat anti-rabbit H & L Alexa Fluor 488 (ab150077), ACE-lysine antibody (ab21623), and mounting medium with DAPI (ab104139) were purchased from Abcam. Antibodies against SENP1 (A1260), Ubc9 (A2193), PIAS2 (A5654, the antigen peptide is specifically from human PIASxα), ATF2 (A0757), and pATF2 (T69/T71) (AP0525) were all purchased from ABclonal (Shanghai, China). Antibodies against mouse M2 FLAG-tag antibody (F1804) and MG132 (SML1135) were purchased from Sigma-Aldrich (St. Louis, MO, USA). Antibody against human Vimentin (9E7E7, sc-66001) was purchased from Santa Cruz (Santa Cruz, CA, USA). Cycloheximide (CHX, 94271) was obtained from Amresco (Solon, OH, USA), and puromycin (P8230) was purchased from Solarbio (Beijing, China). DCFH-DA (50101ES01) and Cell-ROX (50103ES50) were purchased from YEASEN. N-Acetyl-L-cysteine (NAC) (S1623), trichostatin A (TSA) (#S1045), nicotinamide (NAM) (#S1899), and protease inhibitor cocktail (EDTAFree, 100× in DMSO) were purchased from Selleck (Houston, TX, USA). MK2-IN-1 hydrochloride (HY-12834A) was from MCE (Shanghai, China). Pierce^TM^ Protein A/G magnetic beads were purchased form ThermoFisher Scientific (Waltham, MA, USA). Ni^2+^-NTA beads (#30210) were purchased from QIAGEN (Hilden, Germany). All primers synthesis and gene sequencing were conducted by Genewiz (Suzhou, China).

## Generation of MAPK14 knockout gastric cancer cells by CRISPR-Cas9 mediated genome editing

To generate MAPK14 knockout cell lines, sgRNA targeting MAPK14 was cloned into the vector pSpCas9(BB)-2A-GFP (PX458, Addgene). After transfection for 36 h, GFP-positive cells were selected by flow cytometer (Becton Dickinson, Franklin Lakes, NJ), and then subjected single-cell isolation and expansion. MAPK14 knockout and control cell lines were obtained and identified using both Western blot and DNA genotyping analysis.

## Plasmids, site-directed mutagenesis, transfection and lentivirus infection

pLKO.1 puro (#8453, Addgene) lentiviral system was used for shRNAmediated knockdown of SENP1 or Ubc9 in the indicated cells, and siRNA (Genepharma, Shanghai, China) transfection was used for transient knockdown of PIASxα. Mutations of p38α were generated by using the KOD-plus-mutagenesis kit (TOYOBO, Osaka, Japan) and Exnase II enzyme (Vazyme, Nanjing, China) system according to the manufacturer’s instructions. Cell transfection was performed using Hiff-Trans^TM^ Liposomal Transfection Reagent (YEASEN, Shanghai, China) and cell infection was conducted by a lentiviral system. To rescue p38α expression in MGC803/p38α^KO^ and HGC27/p38α^KO^ cell lines, we used a homemade lentivector pGreenPuro-Dual as described previously ^38^.

## Tumor specimens

All human gastric tumor tissues and the corresponding adjacent normal tissues were blindly obtained from surgically resected samples of GC patients in Shanghai Ninth People’s Hospital Affiliated to Shanghai Jiao Tong University School of Medicine. Involved patients consented to participate in this study and consented to publication of the results. This study was approved by Committee of Experiments Research at Shanghai Ninth People’s Hospital, Shanghai Jiao Tong University School of Medicine.

## SUMOylation assays

(1) *Ni^2+^-NTA pull-down assay*: Briefly, HEK293T cells co-transfected with indicated plasmids for 48 h were lysed in Buffer 1 (0.1 M NaH_2_PO_4_, 6 M guanidine-HCL, 0.01M Tris-HCL, 10 mM imidazole, and 10 mM β-mercaptoethanol, pH 8.0). The cell lysates were incubated with Ni^2+^-NTA beads and rotated overnight at 4℃. The beads were collected by centrifugation and successively washed by Buffer 2 (0.1 M NaH_2_PO_4_, 8 M Urea, 0.01 M Tris-HCL, 10 mM βmercaptoethanol, pH 8.0), Buffer 3 (0.1 M NaH_2_PO_4_, 8 M Urea, 0.01 M Tris-HCL, 0.2% Triton X-100, 10 mM β-mercaptoethanol, pH 6.3), and Buffer 4 (0.1 M NaH_2_PO_4_, 8 M Urea, 0.01 M Tris-HCL, 0.1% Triton X100, 10 mM β-mercaptoethanol, pH 6.3). Purified protein was eluted with elution buffer (0.15 M Tris-HCL, 200 mM imidazole, 30% glycerol, 5% SDS, and 0.72 M β-mercaptoethanol, pH 6.7) and determined by immunoblotting. (2) *Immunoprecipitation* *assay*: HEK293T cells transfected with indicated plasmids for 48 h or fresh resected tissue were immediately lysed in 1% SDS lysis buffer (containing 20 mM Nethylmaleimide, 5 mM EGTA, 5 mM EDTA, and a complete protease inhibitor cocktail). The cell lysates were diluted (1:10) in pre-RIPA buffer (250 mM NaCl, 20 mM NaH_2_PO_4_/Na_2_HPO_4_, 0.5% deoxycholic acid sodium salt, 1% Triton X-100), and was incubated with proper Protein A/G beads and M2 FLAG antibody overnight at 4°C. After washing three times with PBS, the samples were subjected to western blotting. The second antibody of IgG fraction monoclonal mouse anti-rabbit IgG (light chain specific) was used for detection of p38α-SUMOylation. (3) *GST pull-down assay*: *E. coli* BL21 transformed by pGEX-4T-1/MAPK14 plasmid with or without the pE1E2S1 construct was induced by 0.2 mM Isopropyl β- d-1thiogalactopyranoside (IPTG) for 12 h at 16℃ and was lysed as per the manufacturer’s instructions (B-PER Protein Extraction Reagent, Thermo Fisher Scientific). Lysate was then incubated with Pierce^TM^ glutathione agarose (Thermo Fisher Scientific) overnight at 4°C. After washing three times with PBS, western blotting was performed to determine *in vitro* p38α-SUMOylation.

## Nuclear/cytosol fractionation assay

Nuclear/Cytosol fractionation kit (Beyotime Biotechnology, Shanghai, China) was used to extract nuclear and cytosolic fractions according to the protocol. Briefly, a total of 3×10^6^ cells were harvested for Nuclear/Cytosol fractionation, and the nuclear and cytoplasmic fraction were separately used for Ni^2+^-NTA pull-down and western-blotting assay with indicated antibodies.

## Immunoprecipitation

Cells were harvested and resuspended in the lysis buffer (150 mM NaCl, 50 mM Tris-HCL, pH 7.4, 1% NP-40, 0.01% SDS, and a complete protease inhibitor cocktail). Cell lysates were incubated at 4°C overnight with Protein A/G mix magnetic beads and specific antibodies. After washing three times with the lysis buffer, the beads were boiled to isolate the protein for SDS-PAGE and immunoblotting.

## Acetylation assays

HEK293T cells were transfected with HA-tagged p38α, p38α^K152R^, or p38α^D150A^ and treated with 4 μM TSA for 12 h and 10 mM NAM for 4 h before harvesting. Cell lysates in lysis buffer (50 mM Tris-HCL, pH 7.4, 150 mM NaCl, 1% NP-40, and a complete protease inhibitor cocktail) were used for incubation with magnetic beads and specific antibodies at 4°C overnight followed by immunoblotting to determine the acetylation level of p38α.

## ROS detection

Briefly, Cells were incubated with 10 µM DCFH-DA probe for 20 min or 5 μM CellROX probe for 30 min at 37℃ after treatment. Next, the cells were washed twice with PBS and immediately imaged on the fluorescence microscope (Olympus, Center Valley, PA). The fluorescence intensity was also measured and analyzed by flow cytometry. A total of 10,000 events/sample were considered for analysis, and ROS levels were recorded in 5 min. Quantitative ROS production compared to control was calculated as the relative medium fluorescence index (MFI).

## Cell proliferation assay

Cell proliferation was measured by using the CCK8 kit (Biotool, Shanghai, China) according to the manufacturer’s procedure. In brief, 2000 cells/well were resuspended in 100 μL complete medium and seeded into a 96-well plate. After culturing at 24 h intervals for 3~4 days, the cells were treated with 10% CCK8 solution and incubated at 37 °C for 1 h. The microplate reader (ThermoFisher, USA) was used to measure the absorbance at 450 nm. Three biological replicates were performed in the experiments and the data were presented as the mean ± SEM.

## Migration and invasion assays

Cell migration and invasion assays were carried out using a Transwell Chamber (Corning, NY, USA) with or without Matrigel (BD Biosciences, MD, USA) coating. Briefly, 5×10^4^ cells/well in serum-free medium were seeded onto the upper compartment of the chamber. Medium containing 10% FBS was placed into the lower compartment as the chemical attractant. After 12~36h incubation, cells migrated or invaded to the outside surface of the filters were fixed in 4% paraformaldehyde, and then stained with 0.01% crystal violet for photograph under a Nikon ECLPISE Ts2R microscope. Positive cells were counted in 5 random fields for statistical analysis.

## Immunofluorescence and confocal microscopy

Cells were allowed to adhere to pre-coated glass coverslips overnight. Next day, they were blocked with 5% bovine serum albumin dissolved in PBS for 1 h after fixing in 4% paraformaldehyde. Cells treated with primary antibody were washed with PBS and then incubated with fluorescence labeled antibodies in PBS for 1 h. After that, Cell nuclei was stained with DAPI for 5 min. Finally, the cells were mounted by using glycerol and randomly observed and captured under the Lecia Tcs SP8 confocal microscope.
