# Supplementary figures and images for: Positive feedback between ROS and cis-axis of PIASxα/p38α-SUMOylation/MK2 facilitates gastric cancer metastasis
Source: Cell Death Dis. 2021 Oct 22;12(11):986. doi: 10.1038/s41419-021-04302-6 (PMC8536665; doi:10.1038/s41419-021-04302-6)

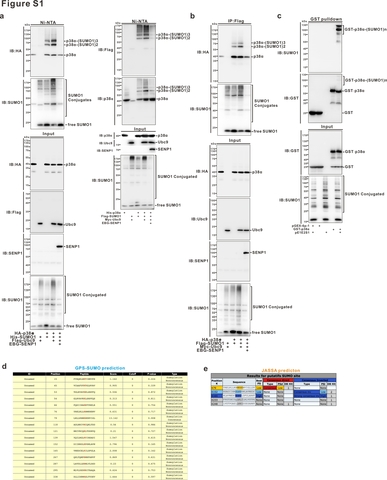

Supplement: Supplementary file 2 — Figure S1 [file 41419_2021_4302_MOESM2_ESM.jpg]

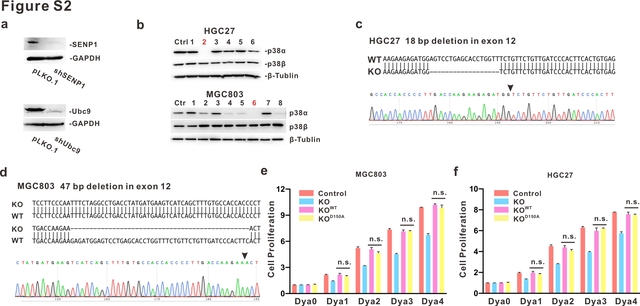

Supplement: Supplementary file 3 — Figure S2 [file 41419_2021_4302_MOESM3_ESM.jpg]

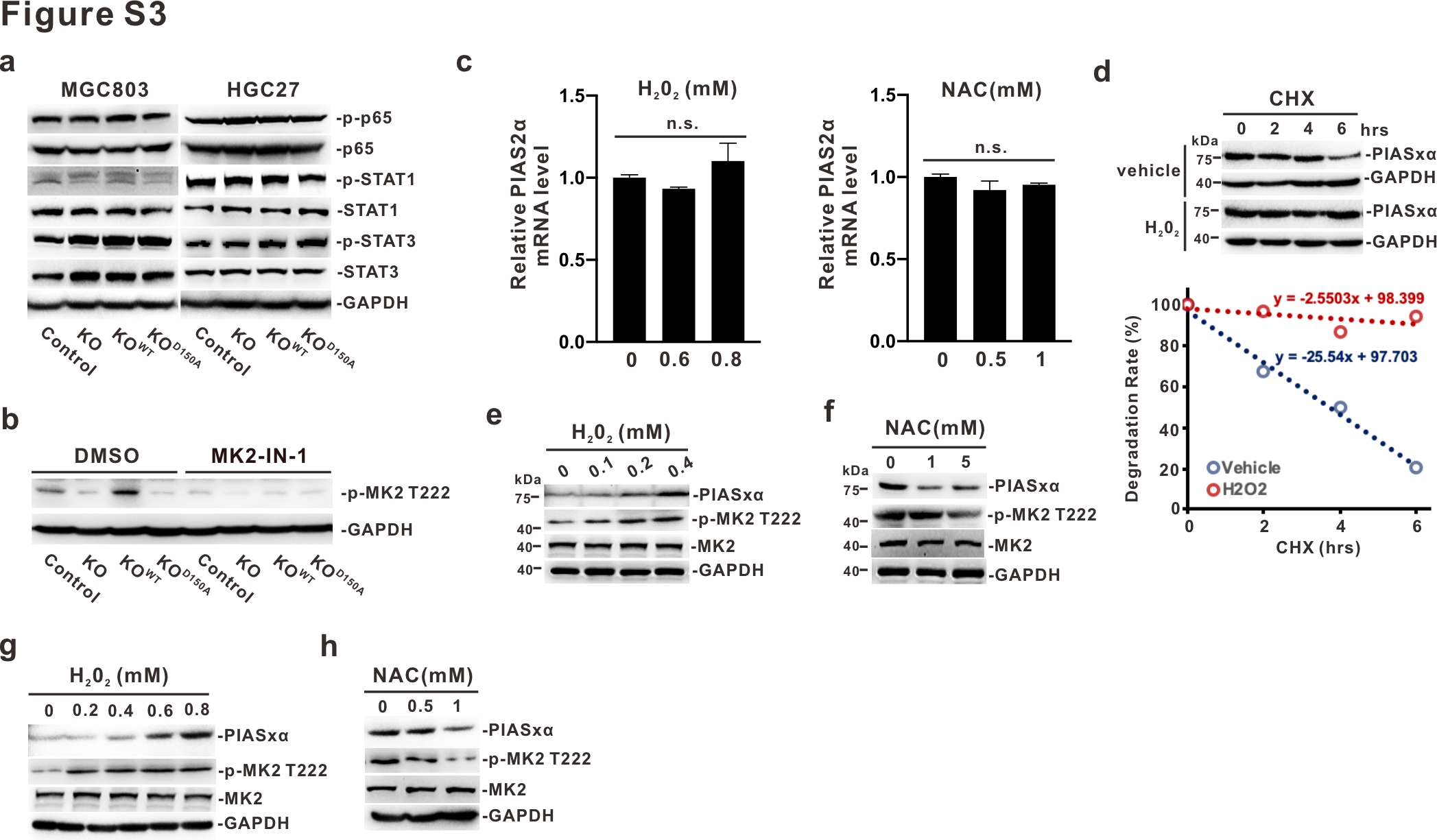

Supplement: Supplementary file 4 — Figure S3 [file 41419_2021_4302_MOESM4_ESM.jpg]

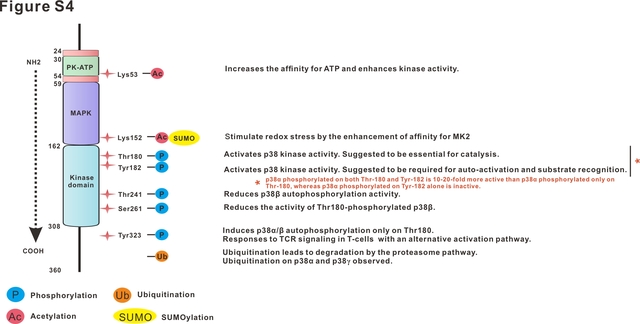

Supplement: Supplementary file 5 — Figure S4 [file 41419_2021_4302_MOESM5_ESM.jpg]
