# Supplementary material for: Positive feedback between ROS and cis-axis of PIASxα/p38α-SUMOylation/MK2 facilitates gastric cancer metastasis
Source: Cell Death Dis. 2021 Oct 22;12(11):986. doi: 10.1038/s41419-021-04302-6 (PMC8536665; doi:10.1038/s41419-021-04302-6)
Supplement: Supplementary file 6 — Supplementary Figure Legends [file 41419_2021_4302_MOESM6_ESM.docx]

## Supplementary Figure Legends

**Fig. S1** **SUMOylation site prediction on p38α.** **(a-c)** The complete membrane information was used for proper data interpretation in Fig.1a, b & c. **(d-e)** Potential SUMOylation sites on p38α were predicted by GPS-SUMO 2.0 Online Service **(d)** and JASSA **(e)**.

**Fig. S2** **Identification of stable cell lines.** **(a)** SENP1 or Ubc9 expression in HeLa cells was abrogated using a lentiviral shRNA system, and knock-down efficiency was validated by western blotting. **(b)** MAPK14^KO^ clones were validated by western blotting in both HGC27 and MGC803 cell lines. **(c-d)** Genotyping analysis was conducted by genomic DNA PCR and Sanger sequencing of No. 2 clone of the HGC27 cell line **(c)** and No. 6 clone of the MGC803 cell line **(d)**. **(e-f)** Cell proliferation of MGC803 or HGC27 cell lines was analyzed by using the Cell Counting Kit8.

**Fig. S3** **Detection of p38-associated proteins.** **(a)** p38-associated proteins were detected in the cell lines generated from both MGC803 and HGC27 by western blotting. **(b)** The indicated cells were treated with MK2-IN-1 for 24 h and western blotting was performed to detect the levels of p-MK2 (T222). **(c)** MGC803 cells were treated with H_2_O_2_ or NAC for 6 h respectively before harvesting, and cells were lysed for real-time RT-PCR analysis. **(d)** MGC803 cells stimulated by H_2_O_2_ or vehicle were treated with 50 μM CHX for indicated times and then cell lysate was subjected to immunoblotting (upper panel). The degradation rate is represented as a line diagram (lower panel). **(e & f)** MGC803 cells were treated with gradient concentrations of H_2_O_2_ **(e)** or NAC **(f)** for 6 h before harvesting, and then cells were lysed for western blotting analysis. **(g&h)** HEK293T cells were treated with gradient concentrations of H_2_O_2_ **(g)** or NAC **(h)** for 6 h before harvesting, and then cells were lysed for western blotting analysis.

**Fig. S4 A schematic model of p38α**. Schematic representation of p38α depicts PKATP-binding (green), MAPK (purple), and kinase domain (blue). Reported posttranslational modification (PTM) sites of p38α and their biological significance are shown on the right side.
